# Harnessing Limestone powder to enhance the thermal crack resistance of manufactured sand

**DOI:** 10.1371/journal.pone.0309105

**Published:** 2024-10-31

**Authors:** Xinting Liu, Jianda Xin, Ying Liu, Zhihao Xiong, Yangbo Li

**Affiliations:** 1 Hubei three gorges polytechnic, Yichang Hubei, China; 2 China institute of water resources and hydropower research, Beijing, China; 3 China Energy Investment, Beijing, China; 4 Shanghai Survey, Design and Research Institute Co., Ltd, Shanghai, China; 5 College of Hydraulic and Environmental Engineering, China Three Gorges University, Yichang Hubei, China; Mirpur University of Science and Technology, PAKISTAN

## Abstract

Manufactured sand concrete (MSC) has increasingly applied in engineering. However, how to enhance the thermal crack resistance of MSC has not clearly studied yet, which limits the wide applications of manufactured sand in engineering. We have adopted the method of mixing limestone powder (LP) to make MSC with four mass fractions (wt.%) of 5%, 10%, 15%, and 20% of LP content, designed the mix proportion of concrete, tested their mechanical properties, observed their microstructure by scanning electron microscopy (SEM) and investigated the effect of LP on anti-cracking performance using temperature stress testing machine (TSTM). These findings reveal that the mechanical properties and thermal crack resistance of 15% LP MSC are the best. Our results also provide a research basis for the MSC to be widely used in engineering.

## 1 Introduction

Concrete is an indispensable building material in such engineering as hydraulic, bridges, roads, and architectures, etc. It has been consumed tremendously and increasingly year by year. For example, the nationwide concrete consumption in 2010 is 2.5 billion cubic meter and the sand consumption is 1.78 billion tons [1]. As one of the main constituent materials of concrete, the sand directly affects the workability, the mechanical properties and durability of the concrete [2–6]. Natural sand is a non-renewable resource in the short term. Such tremendous engineering consumption has led to a gradual shortage of natural sand on earth. Excessive mining of natural sand inevitably results in more and more serious environmental damage, such as riverbank collapse, riverbed subsidence, water quality deterioration, etc. In order to protect natural environment, the manufactured sand is an alternative substitute after the natural sand has been gradually banned [7–12]. The artificial or manufactured sand not only meets the project quality requirement, but also maintain sustainable development. Three Gorges Project adopted manufactured sand to remedy the sand shortage. However, the manufactured sand is quite different from the natural sand in shape. The natural sand is round and smooth, while the manufactured flat and angular, which causes that the MSC is quite different from the natural sand concrete (NSC) in the thermal or mechanical properties. Their size grading also is quite different. The manufactured sand sometimes is lack of super fine particles. So, limestone powder (LP) is mixed into it to improve the strength or thermal crack resistance.

Concrete cracking has always been an nonnegligible problem in engineering [13]. Such factors as strength, elastic modulus, shrinkage, and creep, etc., have a great influence on concrete cracking. Some admixtures, such as fly ash [14], waste granite powder [15], wollastonite ultrafine fiber [16], etc., have been introduced to improve the crack resistance of NSC [17–22]. A set of enhancement mechanism and evaluation methods have been formed. The mechanism lies in the fact that appropriate admixtures are good for particle grading of concrete. The most representative evaluation methods for concrete cracking include the ring method and the flat plate method [23–25], both of which use the time of occurrence of cracks or the area of cracks in concrete specimens as evaluation indicators. Due to the limitation of the specimen size, they are not suitable for the cracking behavior of mass concrete under the action of temperature field in engineering. In order to evaluate the thermal crack resistance of mass concrete more realistically, a new method of evaluation, temperature stress test method [21,26–28], has been proposed by introducing the temperature stress testing machine (TSTM) [28–30]. Adopting this method, such factors as temperature history, degree of restraint [31], different aggregate types [17], different cement types [22], etc., has been analyzed how to affect the thermal crack resistance of NSC.

For MSC, incorporating proper LP [32–34] to enhance the compressive strength [35], flexural and fracture[36], volume stability [37], durability [38,39], shrinkage [23,25] and creep properties [40] of MSC had already been explored. For example, Li found that replacing 10 wt% cement with limestone substantially improved the compressive strength, chloride and water ingress resistance of recycled aggregate concrete due to the decreased pore connectivity [32]. It is also reported that incorporating LP to replace cement can reduce the shrinkage of concrete because LP is less reactive than Portland cement and it can be considered as an inert filler material [35]. It’s worth noting that most research have replaced cement with LP and w/b is thought to be unchanged. On the other hand, to the authors’ knowledge, investigation on effect of LP on thermal crack resistance of concrete has not been studied yet.

Malhotra and Carette [41] studied the effect of stone powder content on the shrinkage of concrete at 217 days at 0.70,0. 53 and 0.40, respectively, and the results showed that the shrinkage of concrete and water ash ratio.And the stone powder content is directly proportional relationship. For the stone powder content of more than 10% of the concrete, the trend of shrinkage is more obvious. The reason for this result is that stone powder accelerates the formation of cement hydration and hydration of carbon and aluminate, and the high content of stone powder increases the dose of water reducing agent in concrete.

Uhcikawa [42] et al. Through the use of limestone powder and several other mineral powder instead of the concrete sand test, that in addition to the filling effect of mineral powder itself, mineral powder sand of concrete cement hydration reaction, hydration products and hardening structure, and mixed cement concrete has no essential difference. Stone powder and other mineral powder instead of sand to prepare concrete increased the number of slurry in concrete, thus increasing the elastic modulus of concrete.

In order to truly evaluate the crack resistance of mass concrete under the joint action of temperature field and strong constraint boundary conditions, a new evaluation method of concrete crack resistance- temperature stress test method is born. This method can simulate the comprehensive influence of various factors on the constraint stress in the actual engineering, and analyze the influence of various factors on the cracking behavior of concrete [43]. After this method was proposed, some scholars [44–48] gradually developed into the current temperature stress testing machine. Many scholars used the temperature stress testing machine to test the cracking of early age concrete, and the test results are more consistent with the cracking behavior of the actual engineering concrete.

Before this paper, some scholars have made some research on the replacement of concrete natural aggregate.To cut down the cement and natural aggregate usage, supplementary cementitious materials including fly ash, rice husk ash and ground granulated blast furnace slag and alternative aggregates, including glass aggregate, rubber aggregate and recycled concrete aggregate are introduced into pervious concrete [49,50].Other scholars have evaluated the potential of Linz-Donavitz slag aggregate as a substitute for natural coarse aggregate in concrete production to promote the sustainable utilization of available resources between steel and the construction industry [51]. And scholars uses steel slag as an aggregate to prepare sustainable concrete [52].

In order to explore the influence of different LP proportions on the anti-cracking performance of MSC, MSC with 5%, 10%, 15% and 20% LP replacing fine aggregate (i.e., manufactured sand) and NSC as the comparison group has been made, which is different from the mix proportions design of MSC in existing literature (i.e., cement was commonly replaced by LP). SEM pictures have been used to observe the microstructure of concrete. TSTM has been utilized to test the stress and occurrence time of crack of all specimens. In order to unveil the underlying mechanism of thermal crack resistance of MSC with LP, the cracking temperature difference and cracking stress of all specimens also have been analyzed.

## 2 Materials and testing methods

### 2.1 Raw materials

**Fig 1:**
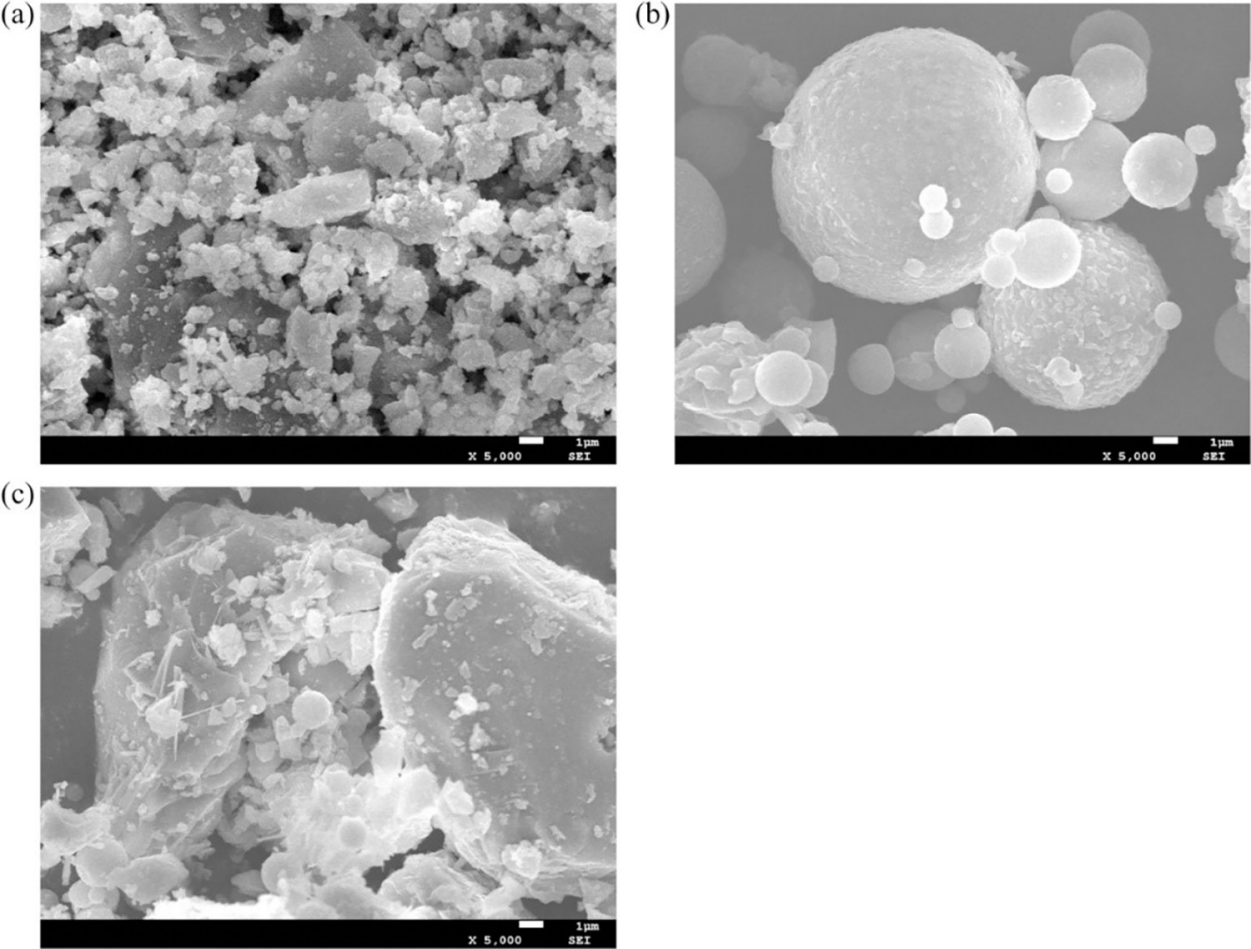
The morphology of concrete aggregates under SEM:(a) The LP between the MSC aggregate gaps, (b) The fly ash and between the MSC aggregate gaps (c) The slag Portland cement between the MSC aggregate gaps.

In this study, the raw materials of the concrete as shown in Fig 1 include P•S•A32.5 Portland slag cement, fly ash (Engineering Class I), LP with the particle size less than 0.0374 mm, big and extra-large stones with the size ranging from 40 to 150 mm, medium stones (the particle size ranges from 20 to 40 mm), small stones (the particle size ranges from 5 to 20 mm), JM-Ⅱ water reducing agent and GYQ air-entraining agent. These stones are mixed in a ratio of 20:20:30:30 (mass fraction) to achieve a bulk density of 1682 kg/m^3^ and a void ratio of 39.5%. The natural sand is river sand with a fineness modulus of 3.09, a saturated surface dry apparent density of 2690 kg/m^3^, and a water absorption rate of 1.4%. The manufactured sand is made of limestone crushed, washed, and screened, with a fineness modulus of 3.72, a saturated surface dry apparent density of 2790 kg/m^3^, and a water absorption rate of 1.1%. The aggregate particle grading curve is shown in S1 Fig, which shows that appropriate LP incorporation improves the particle grading of manufactured sand.

The mix proportion of concrete for the reference "JGJ / T 318–2014 Technical Specification for the application of limestone powder in concrete" is shown in Table 1.TR, JZ5, JZ10, JZ15 and JZ20 represent NSC, 5%, 10%, 15%, and 20% LP of MSC, respectively. The designed strength grade of the concrete is C30 and the water-cement ratio is 0.5. After the concrete is mixed, the big and extra-large stones are wet-screened out to ensure the maximum aggregate particle size is less than 40 mm in the concrete mechanical performance and thermal crack resistance tests.

**Table 1:** Mixture proportions of concrete (kg/m^3^).

	Water	Cement	Fly ash	LP	Manufactured sand	Natural sand	Gravel	Water reducer	Air-entraining admixture
TR	83	108	58	0	0	533	1776	0.996	0.02656
JZ5	82	108	58	26.65	506.35	0	1776	0.996	0.02656
JZ10	83	108	58	53.3	479.7	0	1776	0.996	0.02656
JZ15	85	108	58	79.95	453.05	0	1776	0.996	0.02656
JZ20	88	108	58	106.6	426.4	0	1776	0.996	0.02656

### 2.2 Mechanical performance tests

Although the mechanical properties of concrete cannot directly reflect the strength of its thermal crack resistance, they are also important factors affecting concrete cracking. In order to investigate the influence of LP on the mechanical properties of MSC and the effect on the thermal crack resistance, a series of mechanical parameter tests at 14-day age concrete under different LP contents have been conducted.

We test the tensile strength, compressive strength, elastic modulus and thermal expansion coefficient of concrete with different mix ratio. The tensile strength of MSC and NSC was relatively strong, and the maximum tensile strength of 10% stone powder content increased by 7. 5% relative to the minimum value at 14 days of age. The overall compressive strength also shows that the compressive strength of MSC is relatively strong with NSC, and the influence of stone powder content on the compressive strength basically follows the law of the first increasing and then decreasing. In the same instar, the elastic modulus of NSC is smaller than that of MSC. In 3 days and 28 days, the elastic modulus of concrete decreases with the increase of the content of stone powder, reaching the maximum at 10% stone powder content. At the age of 7 days and 14 days, the elastic modulus of concrete is gradually decreased, and the elastic modulus of concrete decreases to the same extent. After the hardening of concrete, the influence of the line expansion coefficient does not change much with the age, so the line expansion coefficient of the 14 days of the concrete specimen is measured as the data reference. At the 14th instar age, the linear expansion coefficient becomes an increasing trend and the growth rate gradually increases. See Table 2 for specific data.

**Table 2:** Material parameter properties of concrete.

	Elastic modulus E*10^9 (Pa)		Destiny *ρ* (kg/m^3^)		Poisson’s ratio *v*		CTE α (10^−6^°C^-1^)		Compressive strength *f*_c_*10^6 (Pa)		Specific heat capacity *c* *10^3 (J/kg•°C)		Thermal conductivity λ (W/m•°C)		Tensile strength *f*_t_*10^6 (Pa)	
TR		25.2		2325.71		0.21		11.09		16.37		1.004		1.980		1.62	
JZ5		30.9		2301.43		0.19		11.07		18.85		1.048		1.987		1.87	
JZ10		30.7		2340.00		0.22		11.17		19.87		0.987		2.018		2.01	
JZ15		29.2		2362.86		0.20		11.53		19.71		1.029		2.017		1.94	
JZ20		26.9		2395.71		0.17		12.58		18.14		1.043		2.293		1.90	

### 2.3 Thermal stress test

To investigate the influence of the LP content on the thermal crack resistance of MSC, the TSTM is used to carry out the experiment by considering simultaneously the mechanical properties of the concrete, the boundary conditions of temperature, and restraint degree. The TSTM mainly includes four parts: temperature control system, displacement control system, load measurement system and environmental simulation system. During the test, the water circulation and servo motor can truly simulate the change of the temperature field and the restraint degree of the concrete specimen from pouring to hardening. The temperature sensor and the stress sensor will measure the temperature and stress of the concrete throughout the cycle, to effectively evaluate the thermal crack resistance or cracking sensitivity of the concrete. The size of the concrete specimen in the device is 150×150×1500 mm. The temperature control range of the instrument is -20°C~80°C, the accuracy is 0.01°C, the range of the displacement sensor is ±1.5 mm, and the accuracy is 0.001 mm.

After the concrete specimen is cast and vibrated, the heating rod and compressor in the temperature control system change the temperature of the water passing through the formwork, thereby the internal temperature of the concrete is controlled, which makes the concrete shrink or expand. When the deformation value of the specimen reaches the threshold, the displacement control system will be activated to control the tension or compression of the grip at the end of the specimen according to the set degree of restraint. The restraint degree refers to the percentage of the restricted free deformation and free deformation of the concrete. After the concrete is stretched or compressed reciprocally, the stress accumulated inside the concrete becomes larger and larger. Until it is greater than its tensile strength, and the concrete specimen cracks. The temperature sensor and displacement sensor will measure and record the temperature and stress value of the concrete specimen in real time.

All the specimens have been maintained at a constant temperature of 20°C from the beginning to the 14-day age. After 14 days, specimens are cooled under 100% restraint degree until they crack by tension. According to the cooling temperature setting used in the study of the temperature course and crack resistance relationship of concrete by Ding Jiantong [31] et al, we set the cooling rate of concrete to 0.5°C / h and measured the temperature and stress at this cooling rate to determine the cracking sensitivity of MSC with different LP content and the same boundary. The deformation threshold of each loading step is set as 4 μm, which means that as temperature inside the specimens decrease gradually, they will shrink slowly. Until the shrinking displacement on the free chuck is close to 4 μm, the current loading step will pause, and the free chuck will be stretched back to the original length of the specimen. This step will continue to repeat until the specimens crack by tension. The material parameters are listed in Table 2.

### 2.4 Analytic calculations

According to the deformation characteristics of the concrete specimen, the concrete strain composition of the TSTM test under the fully constrained condition is shown in Eq (1). This ratio, as shown in Eq (2), is an important indicator for evaluating the relationship between creep and free strain and relaxation characteristics. In addition, this ratio analyzes the influence of creep factors on the cracking process of concrete, and studies the influence mechanism of LP admixture on concrete cracking. Under different restraint degrees and temperature histories, the cracking temperature difference of the concrete can also be theoretically calculated. According to the strain separation, the relationship between free strain, elastic strain and creep of concrete can be expressed by Eq (3). Assuming *ε*_*free*_ = *αΔT*_1_, the concrete specimens are in rapid cooling, and the time is short (water-cement ratio = 0.5, autogenous volume deformation is negligible), and Eq (2) is substituted into Eq (3) to obtain Eq (4).

$$\Delta \varepsilon_{e}+\Delta \varepsilon_{cr}+\Delta \varepsilon_{free}=0$$ (1)

$$\Psi =\frac{\varepsilon_{cr}}{\varepsilon_{free}}$$ (2)

$$\gamma_{R}\varepsilon_{free}=\varepsilon_{e}+\varepsilon_{cr}$$ (3)

$$\Delta T_{1}=\frac{\varepsilon_{e}}{\alpha \gamma_{R}(1-\Psi )}$$ (4)

Where *Δε*_*e*_ is the elastic strain of concrete specimen, *Δε*_*cr*_ the creep of concrete specimen, *Δε*_*free*_ the free deformation of concrete specimen, *ψ* the ratio of creep to free strain of the concrete specimen, *γ*_*R*_ the restraint degree at both ends of concrete specimen, *ΔT*_1_ the cracking temperature difference of concrete specimen, *α* the CTE.

### 2.5 SEM imaging

In order to expose the effect of LP, the microstructures of NSC and MSC with 5%, 10% and 20% LP are imaged by SEM scanning. The instrument for SEM pictures is JSM-7500F scanning electron microscope produced by Japan Electronics Corporation.

## 3 Results and discussions

### 3.1 Mechanical properties

**Fig 2:**
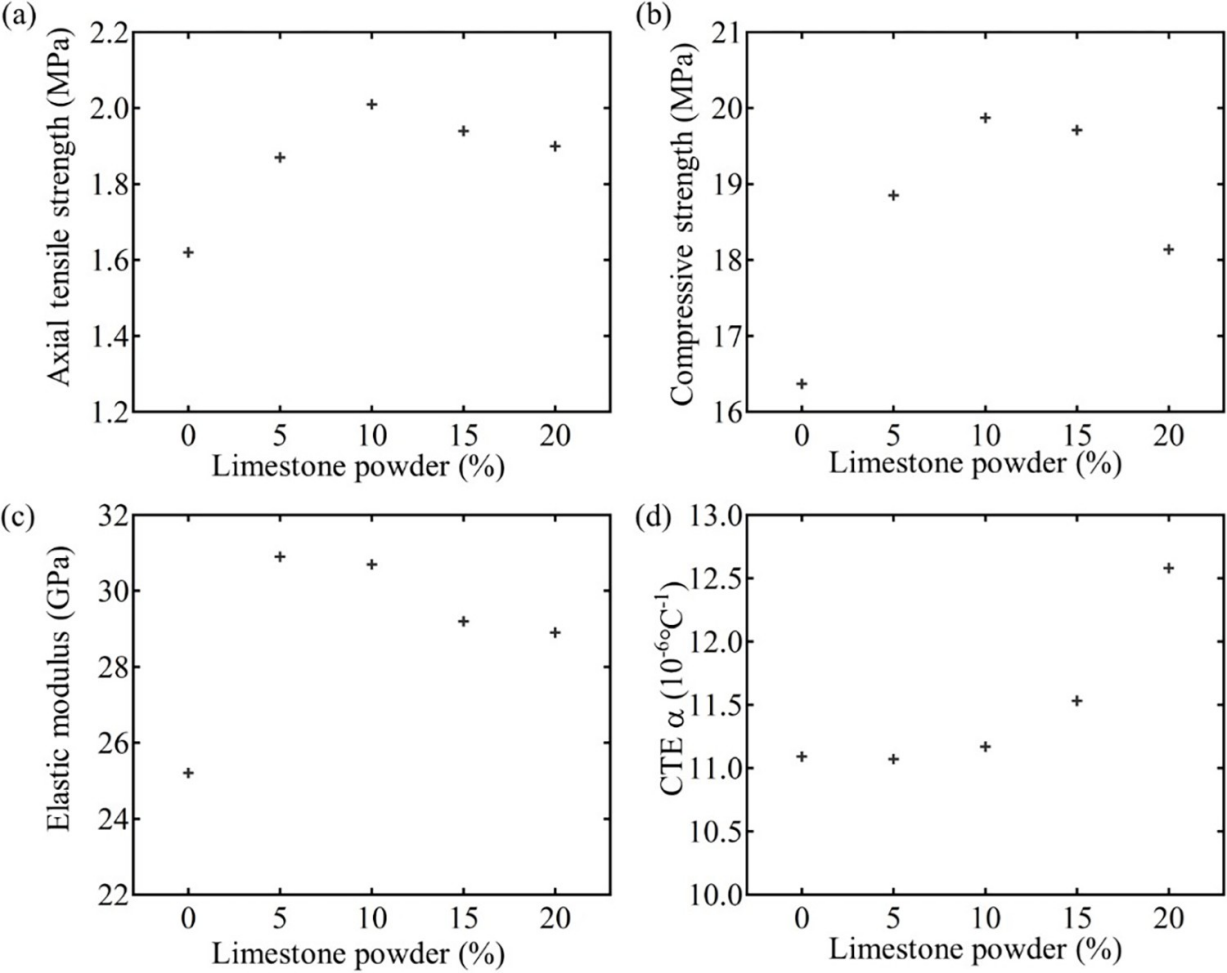
Mechanical performance curves of MSC with different LP proportions include (a) the axial tensile strength, (b) compressive strength, (c) elastic modulus, and (d) CTE. Notes: 0% stands for NSC.

Fig 2 shows that MSC performs mechanical tests over NSC at 14 days of age. Test the tensile strength, compressive strength, elastic modulus and thermal expansion coefficient of concrete with different LP content at 14 days old. The results are shown in Fig 2(A)–2(D). Fig 2(A)–2(C) shows that the tensile strength, compressive strength, and elastic modulus of MSC all show an increase first and then a decrease later. In addition, the tensile strength and compressive strength of MSC reach the maximum at 10%, and the elastic modulus is also shown at the maximum at 5% -10%. Fig 2(D) shows that CTE is gradually increasing at the rate of increase. It can be seen from the results that the effect of moderate LP addition is more obvious on improving the mechanical properties of MSC.

Fig 2 shows that the mechanical properties of MSC are better than those of NSC at 14-day age. It is indicated in Fig 2(A)–2(C) that as the increase of the LP, there is an obvious upward and then decrease trend on the tensile strength, compressive strength, and elastic modulus of MSC. When the proportion of LP is about 10%, the axial tensile strength and compressive strength of concrete reach their maximum value. With the increase of LP content, the elastic modulus of MSC gradually decreases, but it is larger than that of NSC. Fig 2(D) shows the coefficient of thermal expansion (CTE) of MSC gradually increases when the LP increases, and the increasing rate gradually becomes larger. Also, as the LP content is 20%, its CTE is the largest. Abovementioned findings suggest an appropriate amount of LP has a more obvious effect on enhancing the mechanical properties of MSC.

**Fig 3:**
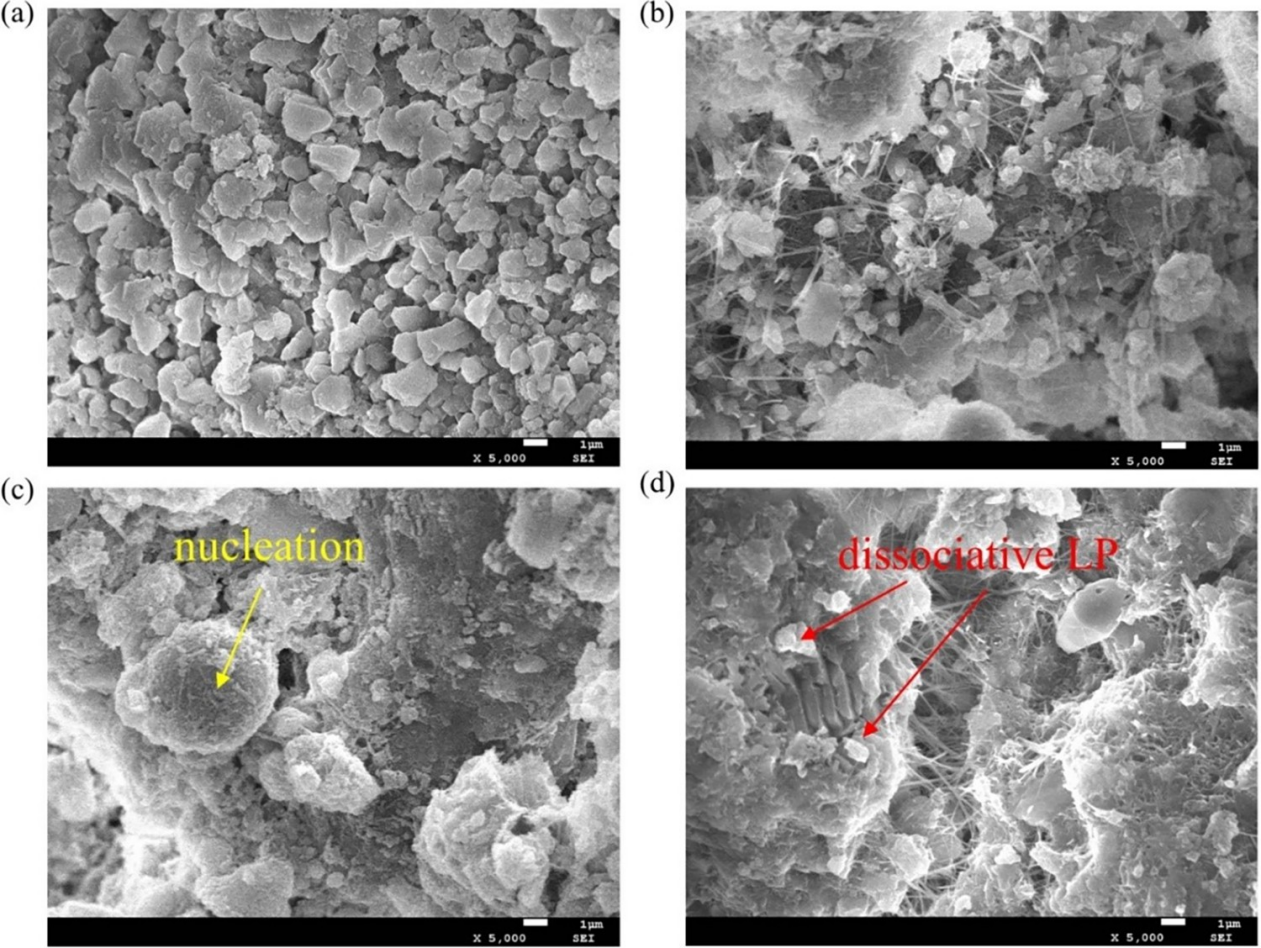
The morphology of (a)NSC, (b)5%, (c)10% and (d)20% under SEM.

The LP content of NLC is 0% and its mechanical properties are lower than MSC. When the LP content is less than 15%, adding LP can refine the structure of cement-based material, optimize the grading of concrete system, and reduce the internal porosity of MSC (see Fig 3). At the same time, it is known from Malhotra and Carette [41] that the addition of LP chemically reacts with chemicals in cement to form aluminate, which can play a role of filling pores, which contributes to the compressive strength of MSC and the increase of CTE. Moreover, according to relevant studies, it can be found that because LP has the effect of promoting cement hydration, the moderate LP content in the early stage promotes the hydration of cement and accelerates the hardening of concrete, which is conducive to the improvement of the tensile strength and compressive strength of MSC. Thus improving the tensile strength, compressive strength and thermal crack resistance of MSC. Secondly, the CTE of MSC increases because the pores in the MSC and the CTE of LP is greater than that of air. At the same time, because of the above mentioned stone powder to promote cement hydration, the compressive strength of concrete also has a positive impact. But when the content of stone powder in concrete is too much, filled between cement and aggregate, because the stone powder itself does not have the cementing effect, so the cementing effect between cement and aggregate is reduced, so as to reduce the mechanical properties of concrete. This shows that the addition of LP can improve the mechanical properties of MSC within the appropriate range.

### 3.2 Micromorphology analysis

The addition of the LP indeed improved the thermodynamic properties of the MSC. The shooting of concrete with different LP content with SEM shows Fig 3, which further verified that the addition of LP played the role of filling the gap between concrete aggregates to accelerate the cement hydration, so as to play the role of improving MSC performance.

Comparing Fig 3(A) with 3(B)–3(D), the LP can fill the pores between the concrete aggregates and improve the compactness of MSC. Fig 3(B) and 3(C) indicate the LP plays the role of crystal nucleus and induces the crystallization of cement hydration products. It can accelerate the hydration of cement and participate in the hydration reaction of cement and enhance the thermodynamic properties of MSC. And with the increase of LP content, the effect is more obvious. However, when the content of LP exceeds a certain limit, as shown in Fig 3(D), excessive LP will not participate in cement hydration reaction, forming dissociative LP, which reduces the performance of MSC and plays a diluting role.

### 3.3 Evaluation of thermal crack resistance of concrete

#### 3.3.1 Evolution of restrained stress and temperature

The cracking temperature of concrete with different LP content was tested. At a specific cooling rate, the temperature of the concrete samples decreases, and the evolution of the constraint stress is closely related to the temperature. The lysis temperature value of MSC changed with increasing LP content, and the results showed that MSC had the best crack resistance when the LP content in MSC was 10% and 15%, respectively.

**Fig 4:**
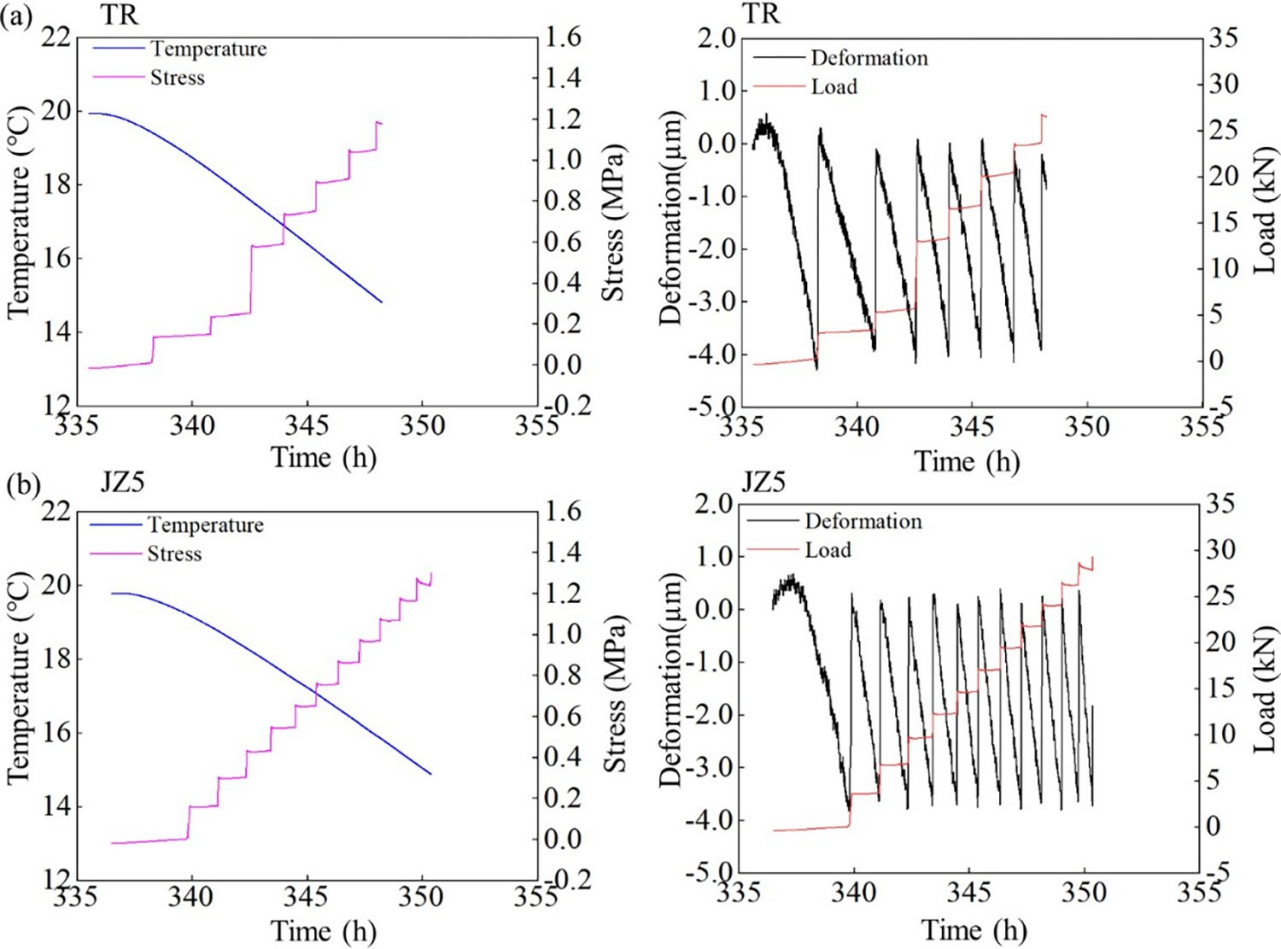
Evolutions of the temperature-time, stress-time, deformation-time, and tension force-time curves of (a) NSC and (b)MSC with 5% LP content under 0.5°C/h cooling rate and 100% constraint condition.

As shown in Figs 4 and 5, the temperature drop phase starts at 336 h and the cooling rate is 0.5°C/h. At the specific cooling rate, the temperature of the concrete specimens decreases, and the evolution of the restrained stress is closely related to the temperature. As the content of LP content increases from 5% to 20%, the cracking temperatures of MSC are 14.89°C, 13.16°C, 12.62°C and 15.81°C, respectively. Under the combined action of elastic modulus and CTE, when the content of LP is 15%, the MSC has the smallest cracking temperature and the largest cracking temperature difference. This shows that the MSC with 15% stone powder content needs a greater temperature change to induce cracking, and has better thermal crack resistance than other test groups. A proper amount of LP content can significantly improve the temperature change resistance of MSC.

**Fig 5:**
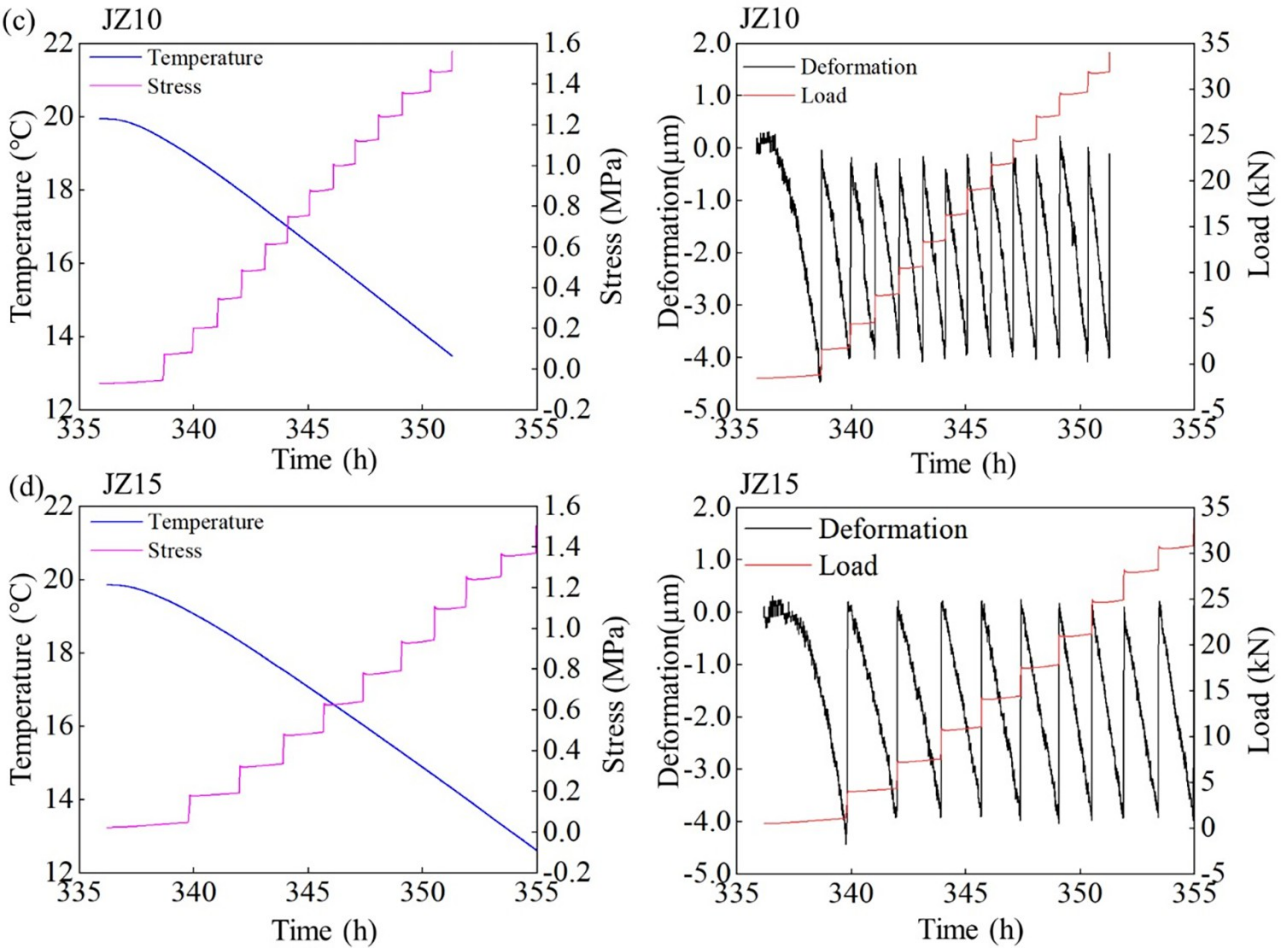
The temperature-time, stress-time, deformation-time and tension force-time curves of MSC with ©10% and (d)15% LP content under 0.5°C/h cooling rate and 100% constraint condition.

As the restrained stress increases, when it exceeds the tensile strength of the concrete specimen, the concrete cracks. The crack stresses of NSC, 5%, 10%, 15% and 20% LP content MSC are 1.2 MPa, 1.32 Mpa, 1.59 Mpa, 1.51 Mpa and 1.35 Mpa, respectively. The cracking stress of NSC is obvious less than that of MSC. The stress of MSC with 10% LP content is the largest when it cracks, which is consistent with tensile strength. The cracking stress of the specimens is lower than the measured tensile strength, which is due to the cumulative damage of the early concrete specimens caused by repeated tension and pressure [26,31]. The ratios of cracking stress to tensile strength of NSC and MSC with 5%, 10%, 15%, and 20% LP content are 0.73, 0.70, 0.78, 0.78 and 0.71, respectively. The larger the value is, the better the thermal crack resistance of concrete has. The results show that when the LP contents in MSC are 10% and 15%, the anti-cracking performance is the best.

#### 3.3.2 Evolution of deformation and tensile force

By studying the relationship between deformation and tension of concrete with different LP content, we can see that LP with 15% content is the best heat resistance and crack resistance in the MSC tested.

Figs 6–8 also imply the evolution of deformation and tensile force. According to the law of free shrinkage and loading of TSTM, when shrinkage displacement evolves from 0 to 4 μm, the specimen is stretched back the original length. This shrinkage results from temperature reduction. Under the same cooling rate, the specimen with larger CTE reaches 4 μm displacement earlier. As a result of multiple shrinkage-stretch cycles, multiple deformation cycles are formed until the cracking of concrete specimens. The variation of tensile force is consistent with that of tensile stress. The greater the tensile force is, the greater the cracking force of concrete specimen is, and the less likely it is to crack.

**Fig 5:**
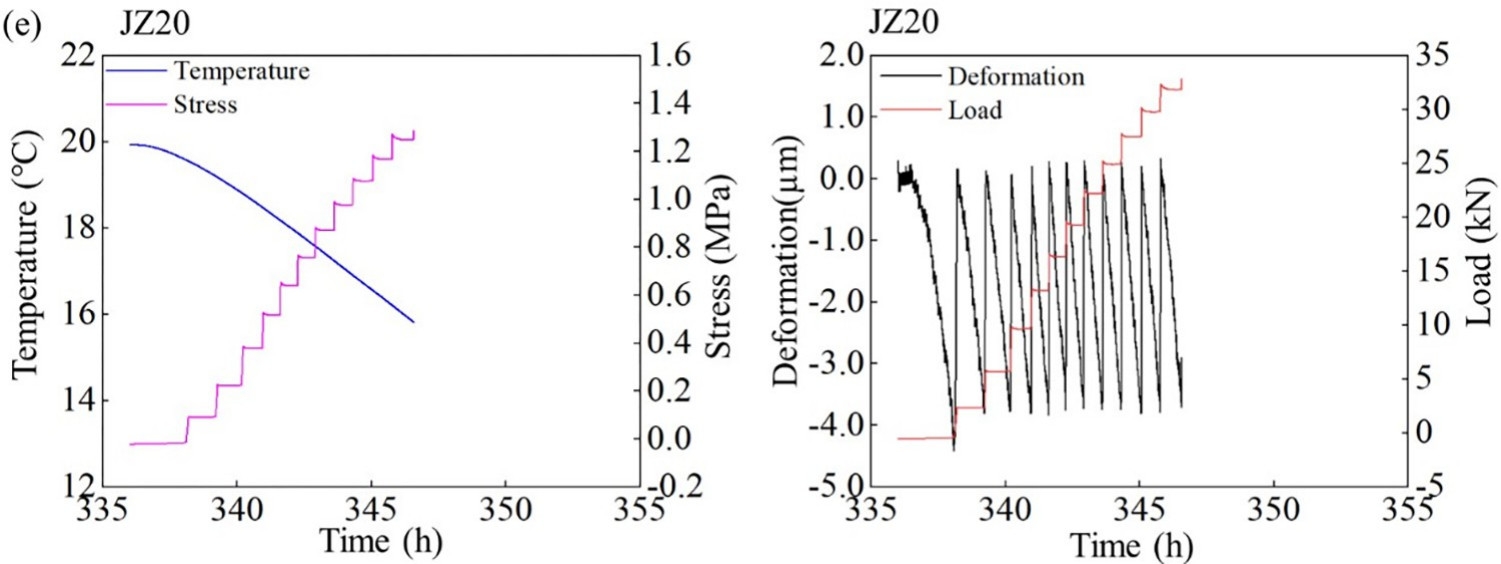
The temperature-time, stress-time, deformation-time, and tension force-time curves of MSC with 20% LP content under 0.5°C/h cooling rate and 100% constraint condition.

**Fig 7:**
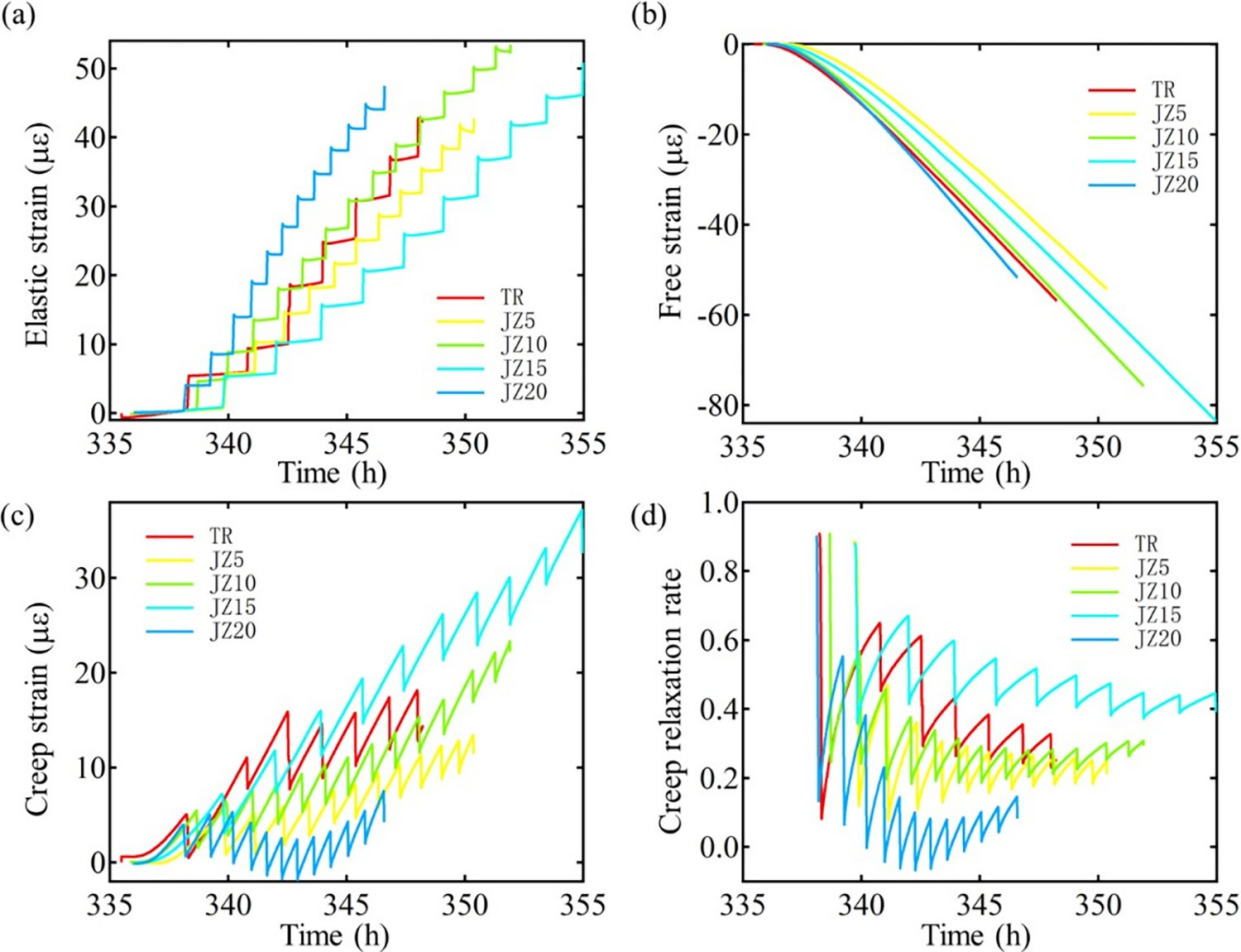
(a)Elastic strain, (b)free strain, (c)creep strain, and (d)creep relaxation evolution curves of different LP proportions of MSC.

**Fig 8:**
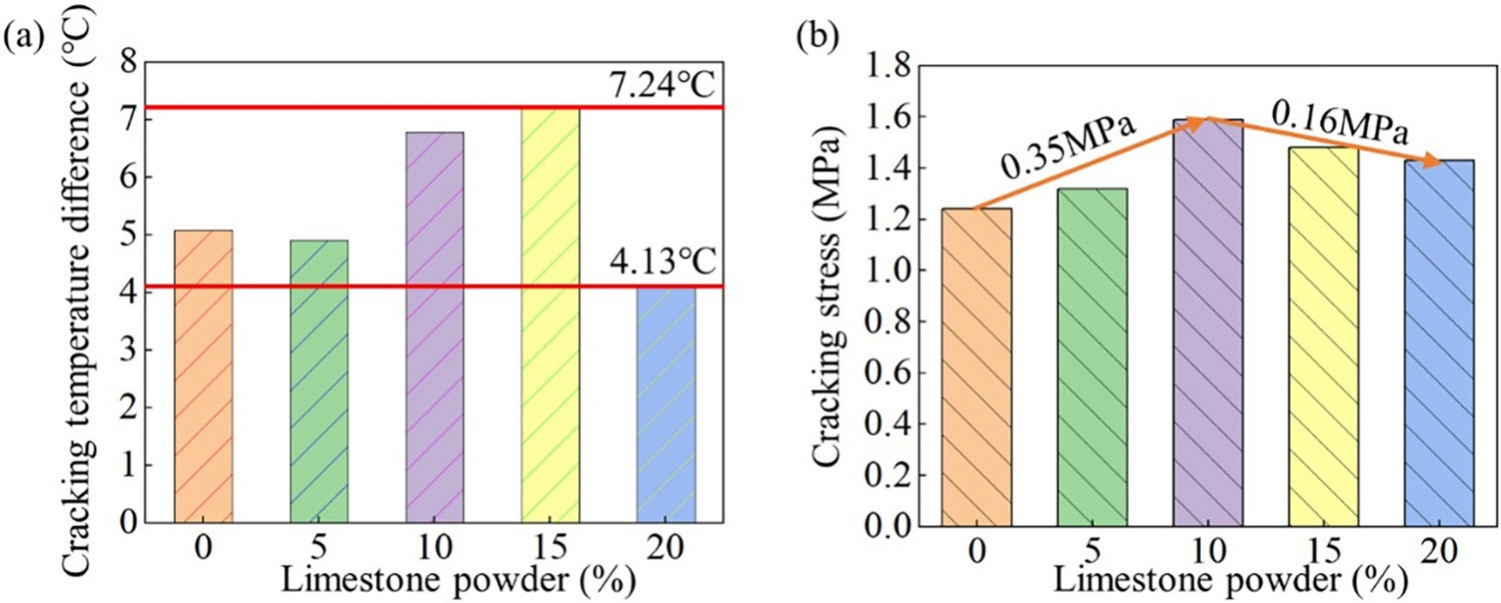
Cracking temperature difference and cracking stress. (a) The relationship between LP and cracking temperature difference of MSC. (b) The relationship between LP and cracking stress of MSC.

Several parameters including cracking moment, cracking temperature, cracking tensile force, cracking stress and free contract-tensile cycle have been introduced to quantitatively describe the cracking resistance of MSC and NSC, as shown in Table 3. The cracking ages of all the concrete specimens are different. MSC with 20% LP is the earliest, and NSC is the second earliest. MSC with 15% LP is the latest, which indicates that the 15% LP MSC has better thermal crack resistance than other test groups. The free shrinkage-stretch cycles of NSC, 5%, 10%, 15% and 20% LP content of MSC are 7, 12, 13, 10 and 12, respectively. In theory, the cycles are proportional to the free thermal strain. Due to 20% LP MSC has largest CTE, it is the one with the earliest cracking and the highest cracking temperature, even though it also undergoes 12 cycles. NSC is the one with the smallest cracking stress and least cycles, which indicates that its thermal crack resistance is less than other MSC. In general, the cracking stress, cracking temperature difference, CTE and elastic modulus meet specific relationship.

**Table 3:** The results of TSTM test.

	Crack age (h)	Crack temperature (°C)	Crack tensile force (kN)	Crack stress (MPa)	Free shrinkage-stretch cycle
TR	348.0	14.80	26.54	1.18	7
JZ5	350.5	14.89	29.32	1.30	12
JZ10	352.0	13.47	34.03	1.57	13
JZ15	355.0	12.62	33.90	1.51	10
JZ20	346.6	15.82	32.83	1.35	12

#### 3.3.3 Evolution of strain

According to Eq (1), the elastic strain, relaxed strain, and free strain of concrete have been separated, and the result are shown in Fig 6. It is obvious that the incorporation of LP does affect the thermal crack resistance of MSC. The elastic modulus and stress of concrete specimens are the direct factors affecting their elastic strain. The free strain is mainly reflected by the CTE of concrete specimens. As the LP increases, the elastic strain (Fig 7(A)) and the free strain (Fig 7(B)) increase. The cumulative elastic strains of NSC and MSC with 5%, 10%, 15%, and 20% LP content are 42.31 με, 42.79 με, 53.45 με, 50.83 με and 47.48 με, respectively, which represent the tensile strengths.

Also, the creep strain of MSC gradually increases. The tensile creep strain starts to develop and follows closely to the evolution of tensile stress. Because the particles of MS are rough and angular, and the length-width ratio is larger than that of NS, the meshing force between particles is strong, which has a certain limitation on deformation. The influence of LP on concrete creep strain is manifold. On the one hand, the LP in MS increases the slurry content in concrete and the possibility of creep. On the other hand, the presence of LP improves the gradation of fine aggregate and reduces the deformation performance of cement. Creep counteracts the tensile stress of concrete and reduces the possibility of early cracking of concrete. The relaxation stress is positively correlated with creep. As the relaxation stress increases, the creep increases and the possibility of concrete cracking decreases. Fig 7(D) indicates that the creep relaxation rates of NSC and MSC with 5%, 10%, 15% and 20% LP content at cracking time are 0.26, 0.21, 0.29, 0.39 and 0.08, respectively. The absolute value of the creep strain of 15% LP MSC is the largest, which greatly reduces the strain of concrete caused by external cooling. Due to the cooling of the ambient temperature of concrete, the contraction state is negative. In order to resist the deformation produced by free strain, the concrete changes to positive value. In the calculation of natural sand concrete and 5%, 10%, 15% stone powder content mechanism sand concrete relaxation rate of the absolute value, with the increase of stone powder, the concrete relaxation rate is gradually increased, 15% stone powder content mechanism sand concrete relaxation rate of the largest, to a large extent reduce the concrete due to external cooling strain. However, the excessive LP in 20% MSC leads to smaller creep relaxation rate of concrete and this is probably because excessive LP played as a role of filler material, which restrict the deformation of matrix. The creep relaxation effect on tensile stress produced by the volume change of external temperature is small, so the concrete is more likely to crack.

#### 3.3.4 Cracking behavior

Fig 8 indicates that the thermal cracking resistance of concrete can be improved by the appropriate amount of LP in MSC. Fig 8(A) shows that when the LP is 15%, the cracking temperature difference of concrete reaches the maximum value, and then decreases. The cracking temperature difference is 5.12°C, 4.90°C, 6.78°C, 7.24°C, and 4.13°C, as the amount of LP increases from 0% to 20%, respectively. The cracking temperature difference of MSC with 20% LP was increases by 75.3% compared with that of NSC. This is due to the high ultimate tensile value, high creep relaxation rate and low elastic modulus of 15% MSC. The cracking stress of MSC is greater than that of NSC, as shown in Fig 8(B). The cracking stresses of NSC and 5%, 10%, 15%, and 20% MSC are 1.18MPa, 1.3MPa, 1.57MPa, 1.51MPa, and 1.35MPa, respectively. The cracking stress increases first and then decreases with the increase of LP in MSC. When the content of LP in MSC is 10%, the cacking stress is the highest.

The concrete cracking stress measured by the TSTM is similar to the change trend of the static tensile strength, but the values are less than the value of the tensile strength. The main reason is that the static tensile strength is measured by statically damage the concrete specimen, while the TSTM is to crack the concrete through the dynamic temperature load under constrained conditions. Under the reciprocating deformation of the temperature load, fatigue of micro-cracks may appear inside the concrete, causing the cracking stress to be lower than the static tensile strength.

The theoretical value of concrete cracking temperature difference calculated according to Eq (4) and the values obtained above is shown in Table 4. There is little difference between the formula of concrete cracking and the experimental results. From the values in the Table 4, the CTE of the concrete specimens gradually increases with the increase of the LP. The elastic strain increases first and then decreases with the increase of LP, and reaches the maximum at 10% LP. The creep relaxation rate also increases first and then decreases with the increase of LP content, and reaches the maximum at 15% stone powder content. As shown in Table 4, the calculation results of the concrete cracking temperature difference are not much different from the test results and the ratios of the calculated value to the measured value are 1.007, 0.999, 0.994, 0.998, 0.993, respectively. The crack resistance of concrete increases first and then decreases with the increase of LP in MSC. Also, MSC with 15% LP has the best thermal crack resistance under the same temperature change.

**Table 4:** Calculation results of concrete cracking temperature difference.

	Crack age (h)	Crack temperature (°C)	Crack tensile force (kN)	Crack stress (MPa)	Free shrinkage-stretch cycle
TR	348.0	14.80	26.54	1.18	7
JZ5	350.5	14.89	29.32	1.30	12
JZ10	352.0	13.47	34.03	1.57	13
JZ15	355.0	12.62	33.90	1.51	10
JZ20	346.6	15.82	32.83	1.35	12

## 4 Conclusions

In order to explore the optimal incorporation content of LP in MSC, NSC and MSC with LP of 5%, 10%, 15%, and 20% have been fabricated. Their deformation and mechanical properties, such as elastic modulus, axial tensile strength, compressive strength, and CTE, have been tested at 14-day age. SEM pictures of MSC with LP of 5%, 10%, 15% and NSC are utilized to analyze the effect of LP directly. In order to test the thermal crack resistance of NSC and MSC with different LP proportions at 14-day age, TSTM has been introduced to monitor the thermal stress as the temperature decreases at the rate of 0.5°C/h. The following conclusions have been drawn according to the results.

1. The elastic modulus is 25.2GPa, 30.9GPa, 30.7GPa, 29.2GPa and 28.9GPa as the amount of LP increases from 0%-20%. And compared with NSC, they have increased by 22.6%, 21.9%, 15.9% and 14.7%, respectively. The elastic modulus gradually decreases with the LP, but the overall value is larger than the NSC
2. The CTE is 11.09×10^−6^/°C, 11.07×10^−6^/°C, 11.17×10^−6^/°C, 11.53×10^−6^/°C and 12.58×10^−6^/°C, respectively, as the amount of LP increases from 0% to 20%. Compared with NSC, they have increased by 0.18%, 0.72%, 4.00% and 13.44%, respectively. As the LP content increases, the linear expansion coefficient of MSC gradually decreases, and the increase gradually becomes larger.
3. SEM images show that LP plays the role of crystal nucleus and induces the crystallization of cement hydration products. LP can accelerate the hydration of cement and participate in the hydration reaction of cement, which enhance the thermodynamic properties of MSC.
4. The creep relaxation rate is 0.26, 0.21, 0.29, 0.39and 0.08, as the amount of LP increases from 0% to 20%. The creep rate first increased and then decreased with the increase of LP content, reaching the maximum value when the stone powder content was 15%, but the excessive LP content (20%) led to the weak MSC creep ability.
5. The cracking temperature difference is 5.12°C, 4.90°C, 6.78°C, 7.24°C, and 4.13°C as the amount of LP increases from 0% to 20%, respectively. The recommended optimal LP content on enhancement of anti-thermal cracking performance of MSC is 15% based on the results in the current research.

## Look into the distance

1. This paper only studies the influence of stone powder content on the crack resistance of mechanized sand concrete in one ratio. Through previous studies, it is concluded that under different water-cement ratio, the content and type of stone powder on the mechanical properties of concrete are also different, and then affect the crack resistance of concrete. Therefore, the influence of stone powder content and type on the crack resistance of mechanical sand concrete.
2. The particle size of stone powder is equal to fly ash, and the incorporation of fly ash will increase the number of slurry in concrete. Therefore, the influence of different fly ash content on the law of crack resistance of mechanized sand concrete also needs to be further studied and analyzed.
3. In this paper, the crack resistance of sand concrete specimens at 14d age was studied. It can also study the crack resistance of various aged concrete specimens, and analyze the development of concrete crack resistance and specific concrete strain under different stone powder content.
4. In the study of the crack resistance of concrete in this paper, only the 100% constraint and the crack resistance under the cooling condition of 0. 5°C / h are considered. However, in the actual engineering, the constraints of each part of concrete are different, and the environmental temperature also changes with the season and weather. Therefore, in order to make the mechanism sand widely used in engineering, it is of great significance to study the crack resistance of the mechanism sand concrete with different stone powder content under different constraints and temperature course.

## Supporting information

S1 Fig Diameter grading curve of natural river sand and mechanism sand. (TIF)

S1 Data (DOCX)

## Abbreviations

MSC: Manufactured sand concrete
LP: Limestone powder
SEM: Scanning electron microscope
TSTM: Temperature stress testing machine
NSC: Natural sand concrete
TR: Natural sand concrete
JZ5: 5% LP of manufactured sand concrete
JZ10: 10% LP of manufactured sand concrete
JZ15: 15% LP of manufactured sand concrete
JZ20: 20% LP of manufactured sand concrete
C30: The compressive strength of each cubic concrete is 30 MPa
CTE: Coefficient of thermal expansion
