# Supplementary figures and images for: Harnessing Limestone powder to enhance the thermal crack resistance of manufactured sand
Source: PLoS One. 2024 Oct 31;19(10):e0309105. doi: 10.1371/journal.pone.0309105 (PMC11527281; doi:10.1371/journal.pone.0309105)

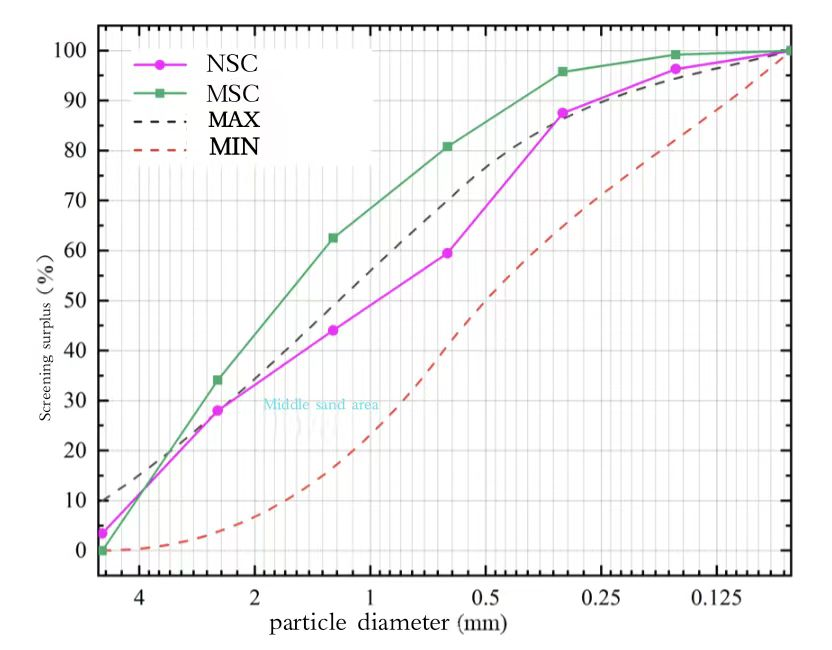

Supplement: S1 Fig — (TIF) [file pone.0309105.s001.tif]
