# Supplementary material for: Harnessing Limestone powder to enhance the thermal crack resistance of manufactured sand
Source: PLoS One. 2024 Oct 31;19(10):e0309105. doi: 10.1371/journal.pone.0309105 (PMC11527281; doi:10.1371/journal.pone.0309105)
Supplement: S1 Data — (DOCX) [file pone.0309105.s002.docx]

**Mixture proportions of concrete (kg/m³)**

|  | Water | Cement | Fly ash | LP | Manufactured sand | Natural sand | Gravel | Water reducer | Air-entraining admixture |
| --- | --- | --- | --- | --- | --- | --- | --- | --- | --- |
| TR | 83 | 108 | 58 | 0 | 0 | 533 | 1776 | 0.996 | 0.02656 |
| JZ5 | 82 | 108 | 58 | 26.65 | 506.35 | 0 | 1776 | 0.996 | 0.02656 |
| JZ10 | 83 | 108 | 58 | 53.3 | 479.7 | 0 | 1776 | 0.996 | 0.02656 |
| JZ15 | 85 | 108 | 58 | 79.95 | 453.05 | 0 | 1776 | 0.996 | 0.02656 |
| JZ20 | 88 | 108 | 58 | 106.6 | 426.4 | 0 | 1776 | 0.996 | 0.02656 |

**Material parameter properties of concrete**

|  | Elastic modulus *E*10^9* (Pa) | | Destiny *ρ* (kg/m^3^) | | Poisson's ratio *v* | | CTE α  (10^-6^℃^-1^） | | Compressive strength *f_c_**10^6  (Pa） | | Specific heat capacity *c* *10^3  (J/kg•℃) | | Thermal conductivity λ (W/m•℃) | | Tensile strength $f_{t}$*10^6  (Pa) | |  |
| --- | --- | --- | --- | --- | --- | --- | --- | --- | --- | --- | --- | --- | --- | --- | --- | --- | --- |
| TR | | 25.2 | | 2325.71 | | 0.21 | | 11.09 | | 16.37 | | 1.004 | | 1.980 | | 1.62 | |
| JZ5 | | 30.9 | | 2301.43 | | 0.19 | | 11.07 | | 18.85 | | 1.048 | | 1.987 | | 1.87 | |
| JZ10 | | 30.7 | | 2340.00 | | 0.22 | | 11.17 | | 19.87 | | 0.987 | | 2.018 | | 2.01 | |
| JZ15 | | 29.2 | | 2362.86 | | 0.20 | | 11.53 | | 19.71 | | 1.029 | | 2.017 | | 1.94 | |
| JZ20 | | 26.9 | | 2395.71 | | 0.17 | | 12.58 | | 18.14 | | 1.043 | | 2.293 | | 1.90 | |

**The results of TSTM test**

|  | Crack age (h) | Crack temperature (℃) | Crack tensile force (kN) | Crack stress (MPa) | Free shrinkage-stretch cycle |
| --- | --- | --- | --- | --- | --- |
| TR | 348.0 | 14.80 | 26.54 | 1.18 | 7 |
| JZ5 | 350.5 | 14.89 | 29.32 | 1.30 | 12 |
| JZ10 | 352.0 | 13.47 | 34.03 | 1.57 | 13 |
| JZ15 | 355.0 | 12.62 | 33.90 | 1.51 | 10 |
| JZ20 | 346.6 | 15.82 | 32.83 | 1.35 | 12 |

**Calculation results of concrete cracking temperature difference**

|  | *γ_R_* | *α* (*με*/℃) | *ε_e_* (*με)* | $\frac{\text{}\text{cr}}{\text{}\text{free }}$ | *ΔT_1_* Calculated | *ΔT_1_*  Measured | Ratio |
| --- | --- | --- | --- | --- | --- | --- | --- |
| TR | 100% | 11.09 | 42.31 | 0.26 | 5.16 | 5.12 | 1.007 |
| JZ5 |  | 11.07 | 42.79 | 0.21 | 4.89 | 4.90 | 0.999 |
| JZ10 |  | 11.17 | 53.45 | 0.29 | 6.74 | 6.78 | 0.994 |
| JZ15 |  | 11.53 | 50.83 | 0.39 | 7.23 | 7.24 | 0.998 |
| JZ20 |  | 12.58 | 47.48 | 0.08 | 4.10 | 4.13 | 0.993 |
